# Glyphosate, but not its metabolite AMPA, alters the honeybee gut microbiota

**DOI:** 10.1371/journal.pone.0215466

**Published:** 2019-04-16

**Authors:** Nicolas Blot, Loïs Veillat, Régis Rouzé, Hélène Delatte

**Affiliations:** 1 Université Clermont Auvergne, CNRS, Laboratoire "Microorganismes: Génome et Environnement", Clermont–Ferrand, France; 2 CIRAD, UMR Peuplements Végétaux et Bio-agresseurs en Milieu Tropical, Pôle de Protection des Plantes, Saint-Pierre, France; University of North Carolina at Greensboro, UNITED STATES

## Abstract

The honeybee (*Apis mellifera*) has to cope with multiple environmental stressors, especially pesticides. Among those, the herbicide glyphosate and its main metabolite, the aminomethylphosphonic acid (AMPA), are among the most abundant and ubiquitous contaminant in the environment. Through the foraging and storing of contaminated resources, honeybees are exposed to these xenobiotics. As ingested glyphosate and AMPA are directly in contact with the honeybee gut microbiota, we used quantitative PCR to test whether they could induce significant changes in the relative abundance of the major gut bacterial taxa. Glyphosate induced a strong decrease in *Snodgrassella alvi*, a partial decrease of a *Gilliamella apicola* and an increase in *Lactobacillus* spp. abundances. *In vitro*, glyphosate reduced the growth of *S*. *alvi* and *G*. *apicola* but not *Lactobacillus kunkeei*. Although being no bee killer, we confirmed that glyphosate can have sublethal effects on the honeybee microbiota. To test whether such imbalanced microbiota could favor pathogen development, honeybees were exposed to glyphosate and to spores of the intestinal parasite *Nosema ceranae*. Glyphosate did not significantly enhance the effect of the parasite infection. Concerning AMPA, while it could reduce the growth of *G*. *apicola in vitro*, it did not induce any significant change in the honeybee microbiota, suggesting that glyphosate is the active component modifying the gut communities.

## Introduction

Glyphosate, or N-(phosphonomethyl)glycine, is as a broad-spectrum herbicide used for weed control. It blocks the plant growth by inhibiting the 5-enolpyruvylshikimate-3-phosphate synthase (EPSPS), an enzyme of the shikimate pathway involved in the synthesis of aromatic metabolites, including aromatic amino acids [1,2]. The EPSPS is present in plants but also in bacteria and fungi, but not in metazoans. Glyphosate is the most sprayed herbicide worldwide, especially under commercial formulations, the best known being Roundup. The frequency and intensity of glyphosate application as well as the surface of treated croplands have been growing constantly, especially since the marketing of Genetically Engineered Herbicide-Tolerant (GE-HT) crops. The wide use of the herbicide has for consequence the contamination of the environment by glyphosate and its metabolites, the most abundant being the aminomethylphosphonic acid (AMPA). AMPA is mainly produced from glyphosate metabolization by soil and saprophyte microorganisms and by second generation of GE-HT plants, but also from other human activities [3–5]. AMPA is more persistent than glyphosate in the environment. Glyphosate and AMPA can be found in all treated plant products but also in non-target plants, in the soil, in surface and groundwaters and even in the atmosphere [2,3]. Since glyphosate and AMPA contaminate water and plant matrices, they can be ingested by animal consumers. For instance, by foraging contaminated pollen, nectar and water, pollinators are exposed to glyphosate and its metabolites.

The European honeybee, *Apis mellifera*, is the most commonly managed pollinator. It is the main pollinator of major crops and thus offers tremendous economic and ecosystem services [6,7]. Honeybees are exposed to a variety of environmental stressors. Among them, pesticides have been suspected to be partly responsible for the colony losses observed worldwide. By gathering resources honeybee foragers bring back to the colony contaminated materials and the entire colony is then chronically exposed to pesticides [8]. Glyphosate has been detected in various hive matrices, including pollen, nectar, honey and honeybee larvae [9–13]. However few data are available concerning the effect of such exposure to glyphosate on the honeybee, and none concerning AMPA.

Despite its low toxicity to arthropods, glyphosate residues may have sublethal effects and affect their fitness [14–18]. In the honeybee, glyphosate or Roundup formulation can induce an increase in lipid peroxidation, a decrease in β-carotene and derived antioxidants, and a reduction in acetylcholine esterase activity [13,19–21]. A chronic exposure to the herbicide can reduce the honeybee learning performance while acute exposure was shown to reduce its short-term memory [22,23]. Glyphosate can also affect the honeybee navigation by increasing the time and path of homeward flight [24]. Nothing is known concerning AMPA, whose toxicity was shown to be low on another arthropod, the crustacean *Daphnia magna* [4].

Once ingested by consumers, glyphosate and AMPA are in contact with the microbial communities of their gut. While glyphosate is known to affect the soil and aquatic microbial communities, its effects on animal gut communities is beginning to gain attention [3]. Indeed glyphosate residues can disturb the gut microbiota of mammals and reptilians and this dysbiosis could partly be due to the variable capacity of bacteria to proliferate in the presence of glyphosate [25–29]. In the honeybee, the impact of a long-term exposure to glyphosate has recently been initiated. In the midgut of emerging honeybees reared *in vitro*, the bacterial beta-diversity was reduced and the abundance of bacterial taxa changed when larvae were fed with a diet containing glyphosate [30]. Motta *et al*. [31] showed that glyphosate also affect the microbiota of adult honeybee workers in the USA. The present work similarly aimed to assess the effect of glyphosate, but also of its main metabolite AMPA, on the installed gut microbial communities of adult honeybee workers in Europe. We thus discussed the results obtained in both studies.

The adult honeybee gut is colonized by microbial communities, almost exclusively bacteria, within the first week following the emergence of the imago [32,33]. The core microbiota, that is common between workers, is relatively stable and simple. It is composed of five major bacterial taxa that are *Lactobacillus* Firm-4 and *Lactobacillus* Firm-5, the gammaproteobacterium *Gilliamella apicola*, the betaproteobacterium *Snodgrasella alvi* and *Bifidobacterium* spp. related to the *Bifidobacterium asteroides* cluster. Among minor taxa, Alphaproteobacteria are the most abundant. We used Quantitative Polymerase Chain Reaction (QPCR) to compare the abundance of these bacterial taxa in response to glyphosate and to AMPA. Bacterial strains were also isolated from honeybee guts to test whether their growth was sensitive to those contaminants.

## Materials and methods

### Honeybees artificial rearing

Experiments were performed on interior workers collected on brood frames from five colonies of the same apiary in Clermont-Ferrand, France. All colonies came from *A*. *mellifera ligustica* Buckfast nuclei (Paul Jungels strain, Naturapi [34]). Each of the five colonies represented an independent replicate. Workers were divided into cohorts of 70 individuals in cages, incubated and fed with sugar syrup, as described elsewhere [35]. In treated conditions, the syrup was supplemented with pure glyphosate (Interchim SS-7701) and/or AMPA (Sigma-Aldrich 324817).

A first experiment was performed on overwintering honeybees (February 2018) that were submitted to (i) no treatment, (ii) a chronic exposure to 1.5 mM glyphosate in their sugar syrup (or 0.21 g/kg according to syrup density), (iii) an infection with spores of the parasite *Nosema*. *ceranae*, (iv) an infection by *N*. *ceranae* and a chronic exposure to 1.5 mM glyphosate. Chronic exposure to herbicides was performed by feeding honeybees *ad libitum* with sugar syrup containing pesticides. The absence of *N*. *ceranae* and *Nosema apis* in the colonies was checked by PCR [35] on 20 honeybees before experiment initiation. *N*. *ceranae* infection was performed one day before experiment initiation, by collectively infecting cohorts of 70 honeybees with a mean of 150 000 spores of the parasite per bee in 2.5 mL of syrup until complete consumption. *N*. *ceranae* spores were obtained according to Vidau *et al*. [36]. Dead bees were removed daily. Feeders were replaced and weighed daily to measure sucrose consumption [35].

The surprisingly strong effect observed on the microbiota by glyphosate led us to repeat the experiment on cohorts of summer honeybees (June 2018) from the same five colonies, but with more concentrations and contaminants: (i) no treatment and chronic exposures to (ii) 1.5 mM (0.21 g/kg) glyphosate, (iii) 7.5 mM (1.08 g/kg) glyphosate, (iv) 1.5 mM (0.14 g/kg) AMPA, (v) 7.5 mM (0.70 g/kg) AMPA, (vi) 1.5 mM glyphosate and 1.5 mM AMPA, (vii) 7.5 mM glyphosate and 7.5 mM AMPA. The chosen concentrations of glyphosate are above most of the ones measured in hives matrices in other studies [9–13]. However they are related to the mean concentrations found in foraged pollen one day (0.47 g/kg) and four days (0.16 g/kg) following an experimental semi-field treatment [11]. Our experiments would thus be related to an unusually persistent exposure to glyphosate.

### Gut DNA purification and PCR quantification of bacterial taxa

Honeybees were sacrificed 15 days after the experiment initiation. The presence or absence of *Nosema* spores in individuals was checked by microscopy (x400). For each experimental condition and replicate, the full digestive tracts of 8 honeybees were dissected on ice, pooled and stored at -80°C. The total DNA of pooled guts was purified as described in Engel *et al*. [37]. QPCR analyses were performed using the primers referenced in S1 Table. All primer pairs targeted the small subunit (SSU) ribosomal RNA encoding gene but allowed the quantification of specific bacterial taxa that were *Bifidobacterium* spp., all *Lactobacillus* spp. or *Lactobacillus* Firm-5 clade only, *S*. *alvi* (two pairs: Neiss-F/Neiss-R and Beta-1009-qtF/Beta-1115-qtR), *G*. *apicola* (two pairs: Gamma1-459-qtF/Gamma1-648-qtR and Gill-F/Gill-R), Alphaproteobacteria, and the whole Bacteria taxon (two pairs: 341F/534R and BAC338F/BAC805R). QPCR reactions were performed in a final volume of 20 μL containing 0.5 volume of 2X Absolute Blue QPCR SYBR Green Mix (Thermo Scientific), 10 pmol of each primer and 5 to 25 ng of total genomic DNA. Negative controls (water) were included in each set of reactions. Treated and untreated samples from a same replicate were always deposited on the same plate and all plates were duplicated. The PCR conditions (CFX96 Real-Time System Thermocycler, BioRad) were 1 cycle at 94°C for 10 min, followed by 40 cycles of 25 s at 94°C, 25 s at 53°C and 25 s at 72°C. Specificity of reactions was checked by analyzing the melting curves of the amplified products. In order to avoid biases due to non-bacterial DNA (*e*.*g*. from the host or the parasite), QPCR data were normalized to the total bacterial DNA content estimated by the mean of the Cycle Quantification (CQ) obtained with 341F/534R and BAC338F/BAC805R primer pairs. The log2 of the ratio of a taxonomic group in a treated condition relative to the untreated one were obtained by subtracting the normalized CQ of control from the normalized CQ of the treated sample at the corresponding season and sampling day. Statistical analyses were performed using PAST software [38]. Non-normal data gave no significant difference using paired Wilcoxon signed rank or Mann & Whitney tests. Paired t-test was performed for normal data and differences were considered significant for *p*-values below 0.05. When a difference was significant, a similar positive or negative effect was observed in all of the five colony replicates.

### Isolation and growth of gut bacterial strains

Single guts from nine interior honeybees were dissected and homogenized in 800 μL of 1X Phosphate-Buffered Saline. After serial dilutions, samples were plated onto Heart Infusion Broth (HIB) medium and MRS medium supplemented with fructose and cysteine [37] and incubated for 48 h at 34°C under low O_2_ and >9% CO_2_ atmosphere using Oxoid AnaeroGen sachets (Thermo Scientific). Colonies were picked up and isolated onto the same medium several times until axenic liquid cultures were obtained, as verified by replating and microscopic observation of Gram-stained bacteria. Thirteen strains were isolated and their genomic DNA was extracted. Their SSU encoding gene was amplified with the Platinum Taq DNA Polymerase (Invitrogen) using a 1:1:2 mix of primers 27f-YM, 27f-Bif and 1492r (S1 Table). The amplification products were cloned into pGEM-T Vector (Promega). Following transformation and blue/white screening, colonies were cultured and their DNA extracted. The amplicon sequences were obtained using T7 and M13 primers (Eurofins Genomics) and deposited, after the trimming of vector and primer sequences, in GenBank database (accessions MH782109 to MH782121).

Cultures of seven selected isolates were diluted 50 times in fresh HIB medium, but supplemented MRS medium for *Lactobacillus* strains (S2 Table). 750 μL of the dilution were deposited in wells of a 24-well plate containing various quantities of glyphosate and AMPA. Six independent cultures were performed for each herbicide concentration. After 48h of incubation as above, the optical density (OD) of the cultures were measured at 600 nm, using sterile medium as a blank. For statistical analyses [38], Student t-test was performed for normal data otherwise the Mann & Whitney test was performed. Differences were considered significant for *p*-values below 0.05

## Results

Interior overwintering honeybee workers were exposed to 1.5 mM of glyphosate in their feeding *ad*. *lib*., to spores of the parasite *N*. *ceranae*, or to both stressors. To verify the effect of glyphosate on the microbiota, a second experiment was performed on summer honeybees that were chronically exposed to 1.5 mM and 7.5 mM of glyphosate, of AMPA and of stoichiometric mixtures of both xenobiotics.

### Glyphosate and AMPA were not lethal and did not alter food consumption

Survival analyses demonstrated that glyphosate but also AMPA, alone or together, did not induce any significant decrease in honeybee survival, compared to control, whatever the season (S1 Fig). In contrast a significant decrease of survival was observed in *Nosema*-infected bees as previously observed [39]. No synergistic effect was observed between glyphosate and *N*. *ceranae* or between glyphosate and AMPA. The constant decrease on survival in summer could be associated with the shorter lifespan of honeybees during the beekeeping season [40]. The data confirmed previous studies showing that glyphosate is no bee killer [11,13,19,21–23] and showed that it is also true for AMPA. Thus, whatever the observed effects on the microbiota, they ought to be sublethal.

A decrease in food consumption by glyphosate-treated honeybees was suggested but not always observed in previous studies [21,23]. Here, the daily sugar consumption was affected neither by glyphosate, nor AMPA, nor *N*. *ceranae* (S2 Fig). The daily mean consumption of glyphosate and AMPA were between 49.2 and 57.4 nmol.d^-1^.bee^-1^ and between 245 and 264 nmol.d^-1^.bee^-1^ for exposures at 1.5 mM and 7.5 mM respectively. The oral and topic LD50 of glyphosate are far greater than 0.1 mg of active ingredient per bee. Zhu *et al*. [41] estimated the topic LD50 as 3.5 × 10^31^ μg/bee, *i*.*e*. about half the mass of the moon of glyphosate per bee. The daily and cumulative consumptions of glyphosate were thus clearly far below lethality.

#### Glyphosate, but not AMPA, altered the abundance of the major honeybee gut bacteria

The honeybee gut microbiota can be altered by fungicides and acaricides [42]. We tested whether it can be affected by the herbicide glyphosate or by its metabolite AMPA. The relative abundance of the major bacterial taxa of the microbiota, normalized to the total bacterial DNA, was assessed by QPCR after 15 days of chronic exposure. Two-way ANOVA showed significant interactions neither between season and glyphosate treatment, nor between glyphosate and *N*. *ceranae*, nor between glyphosate and AMPA. Multivariate analyses suggested that season and glyphosate, but not AMPA were the two most important components explaining the observed variances, with *S*. *alvi* as the major factor explaining the effect of glyphosate (S3 Fig).

**Fig 1:**
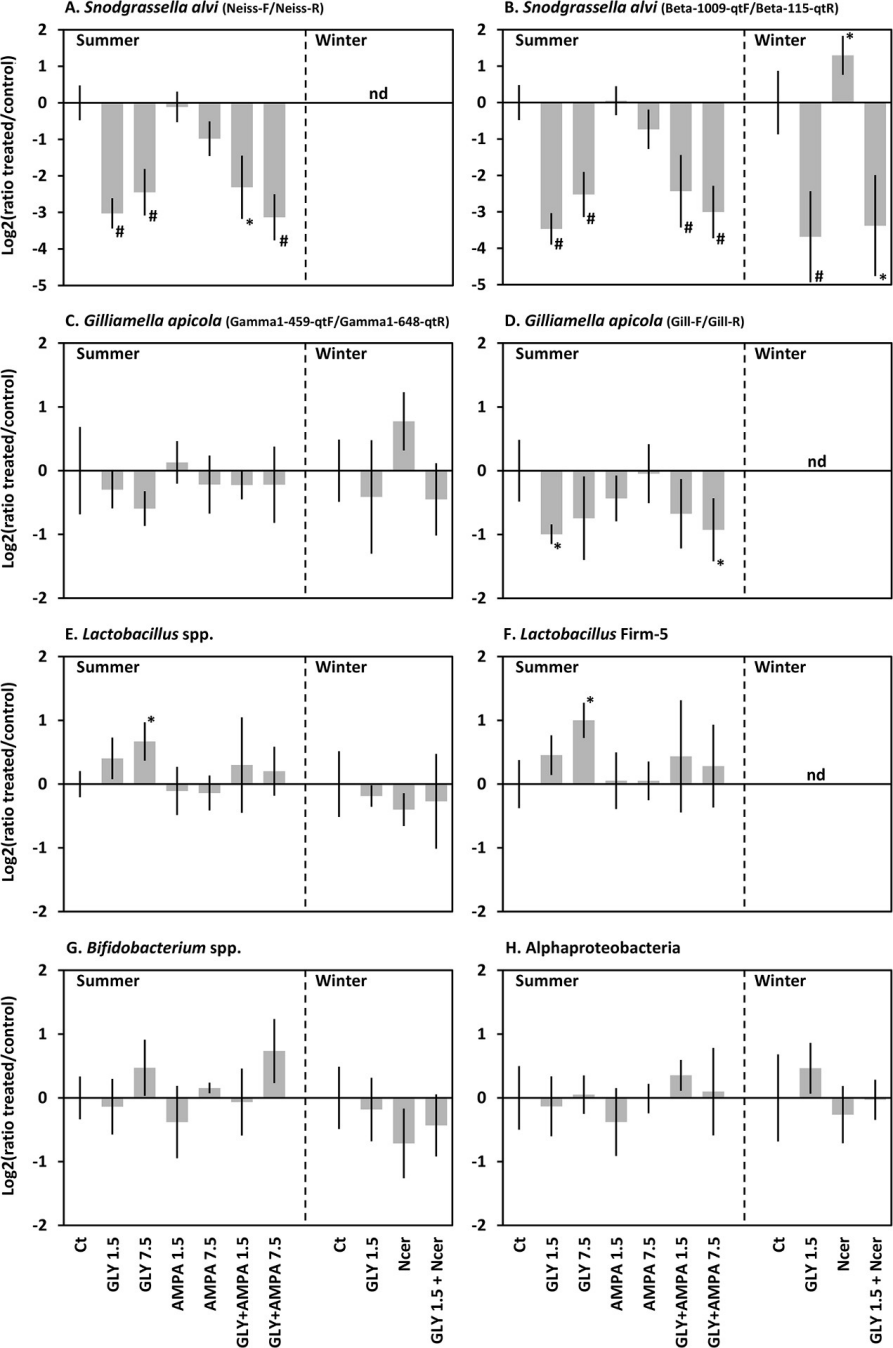
Abundances of the main bacterial taxa of the honeybee microbiota after chronic exposure to glyphosate and/or AMPA, relative to untreated controls. Summer and winter honeybees were chronically exposed to 1.5 mM or 7.5 mM of glyphosate (GLY) and/or AMPA in their feeding sugar syrup. Some bees were also infected with spores of *N*. *ceranae* (Ncer). Control bees (Ct) were not exposed to any stressor. 15 days after treatment initiation, the DNA from guts was extracted and the 16S rDNA of the major taxa of the microbiota was quantified by QPCR and normalized to the total bacterial 16S content. The data are represented as the log2 of the ratio of the abundance in treated condition relative to untreated controls. Data were gathered from five independent colony-replicates. Bars represent the 95% confidence intervals. Stars and hash marks indicate significant differences (paired t-test, *p*<0.05 and *p*<0.005 respectively). When a difference was significant, a similar effect was observed in all the five colony replicates. nd: not determined.

Indeed a very strong and significant decrease of *S*. *alvi* was observed in response to glyphosate, with 5 to 13 times less *S*. *alvi* in treated samples than in untreated controls (Fig 1). This decrease was independent of the applied dose, the season and the QPCR primer pair. Other major taxa of the microbiota were less affected by glyphosate. The relative abundance of *G*. *apicola* was significantly decreased in the presence of glyphosate using Gill-F/Gill-R but not G1-459-qtF/G1-648-qtR primers. A significant increase of all *Lactobacillus* spp. and of *Lactobacillus* Firm-5 only was observed in response to 7.5 mM but not to 1.5 mM glyphosate. The data cannot determine if all *Lactobacillus* spp. are affected or if their global increase is due to the sole Firm-5 clade. At last *Bifidobacterium* spp. and Alphaproteobacteria were not affected.

In contrast to glyphosate, AMPA alone had no significant effect on any bacterial taxa and no interaction between the two molecules was observed following co-exposure.

### Glyphosate did not potentiate *Nosema ceranae* infection

To test whether a glyphosate exposure could increase the effect of an infection by the intestinal parasite *N*. *ceranae*, overwintering honeybees were co-exposed to spores of the parasite and to glyphosate. No stronger effect was observed on mortality (S1 Fig), sugar consumption (S2 Fig) and bacterial abundances (Fig 1) when both the parasite and the herbicide were applied.

### Glyphosate and AMPA can directly affect the growth of honeybee gut bacteria

**Fig 2:**
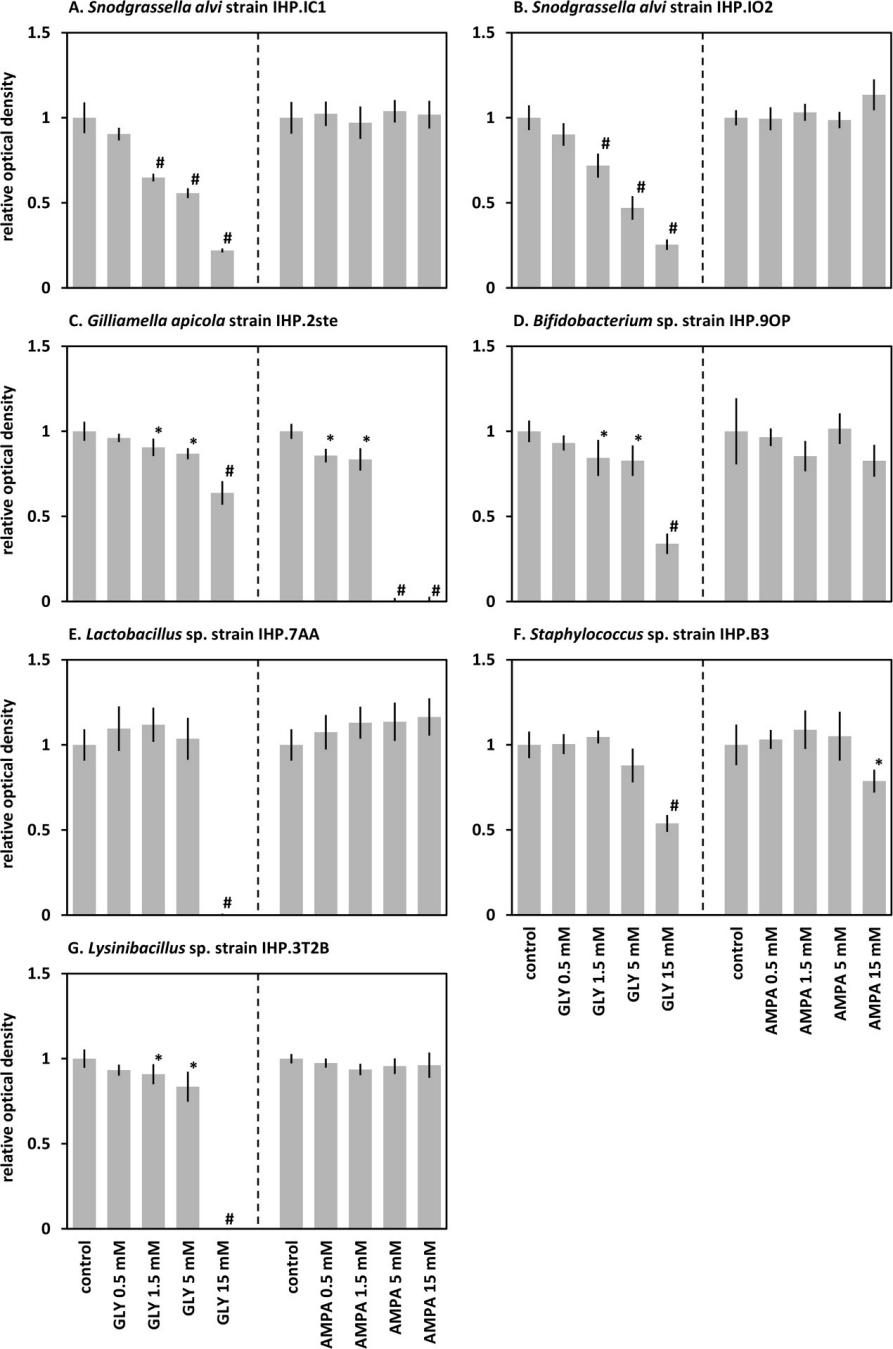
Growth of bacterial strains isolated from the honeybee gut in the presence of glyphosate and AMPA. Strains were inoculated in HIB or MRS medium in the presence of increasing concentrations of glyphosate (GLY) or AMPA. After 48h of growth under anaerobic conditions, the optical density of the cultures was measured at 600 nm. The results are presented as the mean ratio between treated culture and control culture without xenobiotic that was used as a calibrator. Data were gathered from six independent replicates. Bars represent the 95% confidence intervals. A significant difference (Student t-test or Mann-Whitney test) is indicated by a star (*p*<0.05) or a hash mark (*p*<0.001).

In order to test whether honeybee gut bacteria were sensitive to glyphosate and AMPA, strains were isolated from the guts of nine untreated honeybees. Thirteen strains were isolated and identified by the cloning and sequencing of their SSU RNA encoding gene (S2 Table). All obtained sequences were related to known sequences of the honeybee microbiota [43]. Strains belonging to the core gut microbiota taxa were isolated, including *G*. *apicola*, *S*. *alvi*, *Bifidobacterium* sp. and *Lactobacillus* sp.. Neither a *Lactobacillus* Firm-4 nor a *Lactobacillus* Firm-5 strain was isolated but two *Lactobacillus kunkeei* strains. Although the Firm-4 and Firm-5 clades are dominant in the microbiota, *L*. *kunkeei* is known to be the most easily recovered *Lactobacillus* species in cultures [37]. Strains from rare taxa, belonging to the genera *Staphylococcus*, *Hafnia* and *Lysinibacillus*, were also isolated. Seven strains were selected to test the sensitivity of their growth in the presence of increasing concentrations of glyphosate and AMPA (Fig 2).

The growth of *S*. *alvi* was very significantly reduced in the presence of glyphosate but not in the presence of AMPA. Moreover the impairment of *S*. *alvi* growth seemed dose-dependent (S4 Fig). The isolated strain of *G*. *apicola* was also sensitive to glyphosate, to a lesser extent. The growth of *G*. *apicola* was also significantly reduced in the presence of AMPA, and completely impaired in the presence of 5 mM AMPA. The *Bifidobacterium* sp. strain was also sensitive to glyphosate but not to AMPA. In contrast the isolated strains of *Lactobacillus*, and *Staphylococcus* were more resistant to the herbicide and its metabolite.

## Discussion

The present study confirmed the work of Motta *et al*. [31] by showing that glyphosate, although not lethal for the honeybee, can alter its gut microbiota. In relative abundance, a strong decrease in of *S*. *alvi* and an increase in *Lactobacillus* spp. were observed in response to glyphosate (Fig 1), which was consistent with the data of Motta *et al*. [31] in newly emerged and interior bees. In contrast, opposite results were observed on *G*. *apicola*. In absolute abundance, Motta *et al*. [31] showed that glyphosate induced a reduction in the total bacterial content of the honeybee gut microbiota, as well as a decrease in *S*. *alvi*, *Lactobacillus* spp. and *Bifidobacterium* spp..

### Glyphosate altered the microbiota by impairing the growth of some gut bacteria

The growth of axenic cultures *in vitro* showed that glyphosate reduced the generation time of *S*. *alvi* and *G*. *apicola* [31] as well as the biomass production of *S*. *alvi* and *G*. *apicola* and *Bifidobacterium* sp. (Fig 2,[31]). In contrast, the growth of *Lactobacillus* and other Firmicutes strains, including *Staphylococcus* and *Lysinibacillus* (Fig 2), as well as *Bartonella apis* [31] was not altered by glyphosate. These differences may be related to the sensitivities of the EPSPS, the enzyme that is inhibited by glyphosate. Three classes of EPSPS have been characterized by experimental, structural and molecular analyses [44]. The presence of conserved amino acids and the phylogenetic analyses of EPSPS showed that the enzymes of *S*. *alvi*, *G*. *apicola* and *Bifidobacterium* spp. belong to the glyphosate-sensitive Class Iα, and that the enzymes of Firmicutes and *Bartonella* belong to the glyphosate-resistant Class II (S5 Fig). The sensitivity of *S*. *alvi*, *G*. *apicola* and *Lactobacillus* spp. EPSPS was confirmed by gene complementation in *E*. *coli* [31]. Altogether, these data strongly suggest that glyphosate alters the honeybee gut microbiota by directly affecting the growth of species producing a glyphosate-sensitive EPSPS, especially *S*. *alvi*.

#### *G*. *apicola* may comprise distinct populations with different sensitivities to glyphosate

The relative abundance in *G*. *apicola* in response to glyphosate showed contrasted results between the partial decrease observed in the present experiment and the increase observed in the study conducted by Motta *et al*. [31]. Interestingly our results differed according to the primer pairs used for quantification. The abundance of *G*. *apicola* did not change using the G1-459-qtF/G1-648-qtR primers while it decreased using Gill-F/Gill-R (Fig 1). A phylogenetic analysis of *G*. *apicola* 16S sequences found in *Apis* species showed that the two primer pairs targeted different clades of *G*. *apicola* strains (S6A Fig). No *G*. *apicola* sequence derived from *Apis mellifera* microbiota was found within the clades recognized by Gill-F/Gill-R, whereas amplification occurred using these primers. This suggested that the honeybee microbiota caries those targeted strains and that available sequence data do not depict the true diversity of *G*. *apicola*. *G*. *apicola* might comprise distinct phylotypes with different susceptibility to glyphosate. The contrasting increase in relative abundance of *G*. *apicola* observed by Motta *et al*. [31] suggests that the phylotype richness may be even more composite, with variations that might depend on locations, landscape and/or genetic background [45,46]. In response to the herbicide, *G*. *apicola* would experience a complex rebalancing of its phylotypes content.

The origin of the variation of *G*. *apicola* sensitivity toward glyphosate should be related to other adaptations than resistance of the EPSPS. Mechanisms of resistance to glyphosate, including the efflux and the enzymatic degradation of the herbicide, have been described in microorganisms isolated from contaminated soils and streams [3,47]. However the presence of genes encoding phosphonate-degrading enzymes or transporters in a genome would not allow any assertion upon glyphosate resistance as the specificity of these proteins toward different phosphonate molecules, including glyphosate, may greatly vary.

Concerning *S*. *alvi*, the two primer pairs used for quantification gave similar results. This could be explained by their similar specificity, except for one clade that did not comprise taxonomic units from *A*. *mellifera* anyway (S6B Fig). Motta *et al*. [31] did not mention clades responding differently to glyphosate. Thus, the whole *S*. *alvi* species could be affected by the herbicide.

### Gut communities may be indirectly affected through ecological niches alteration

As expected, the growth of strains producing a glyphosate-resistant EPSPS were not affected by glyphosate (Fig 2). Glyphosate even induced an increase in the relative abundance of *Lactobacillus* spp. (Fig 1). Similarly, Motta *et al*. [31] observed a reduced absolute abundance of *Lactobacillus* spp. but an increase in relative abundance of *Lactobacillus* Firm-5 in one experiment on interior honeybees and of *Lactobacillus* Firm-4 in one experiment on newly emerged workers. Several not exclusive hypotheses may be proposed to explain this positive impact of the herbicide.

Motta *et al*. [21] suggested that the dependency of *Lactobacillus* spp. for amino acids released by other bacteria, such as *S*. *alvi*, could explain the absolute negative effect of glyphosate on both species. Such metabolic interactions between gut bacterial species were suggested by genomic data [33]. The microbiota could thus be depicted as a consortium of symbiotic species that faces as a whole its environmental stressors. However experimental data are missing to demonstrate the strength of these interactions.

The observed relative increase in *Lactobacillus* spp. may also be explained by experimental procedures since the use of total bacteria as a reference implies that a relative decrease in some species ought to be balanced by an increase in other taxa. A parallel but ecological hypothesis can be proposed. The reduction of some bacterial species could release ecological niches that would be occupied by others. *S*. *alvi* is thought to form a layer on the ileum wall, itself covered by a layer of *G*. *apicola*, whereas *Lactobacillus* spp. are abundant in the lumen [48]. *Lactobacillus* spp. could benefit from changes in this biofilm-like organization in stressed honeybees by occupying the freed space.

Modifications in the microbiota could also be partly due to a global alteration in the gut homeostasis, keeping in mind that this homeostasis is itself regulated by the microbiota. In *Drosophila melanogaster*, a glyphosate-based formulation can induce a reduction in reactive oxygen species (ROS) and an increase in antioxidant defenses, suggesting that the insect has to cope with an oxidative stress [16]. Such stress may occur in the honeybee as higher lipid peroxidation and lower carotenoids content were observed following an exposure to glyphosate [20]. In mammals, glyphosate is also known to induce detoxification systems and to change the pH of intestinal content [49–51]. Bacteria are diversely sensitive to those environmental conditions and such changes in their habitat could differently impact the bacterial taxa of the honeybee gut.

### Glyphosate may sensitize the honeybee to some but not all pathogens

The gut microbiota is known to protect the host against pathogens through the stimulation of the immune system and the competition for niche occupation [52–54]. Thus gut pathogens may opportunistically benefit from the alterations of the microbiota.

It was shown that the suppression of gut bacteria increases the honeybee susceptibility to the intestinal parasite *N*. *ceranae*, while a supplementation with *Bifidobacteria* and *Lactobacilli* seemed to affect the reproduction success of the parasite [55–56]. We tested whether glyphosate could potentiate the infection by *N*. *ceranae*. Glyphosate did not enhance the effect of the parasite on the honeybee mortality (S1 Fig), sugar consumption (S2 Fig) and gut bacterial abundances (Fig 1). This result contrasted with the higher susceptibility of glyphosate-treated honeybees toward an infection with *Serratia marsescens* [31]. The effect of glyphosate on the susceptibility of the honeybee to pathogens may thus depend on several parameters, such as, the type of pathogen, its sensitivity to the immune and detoxification systems, its capacity to compete with the normal gut flora and its sensitivity to glyphosate. Moreover, the sensitivity of the honeybee to pathogens also depends on a variety of stressors and laboratory experiments are themselves stressful conditions [45].

Interestingly glyphosate may alter the growth of bacterial pathogens. *Paenibacillus larvae* and *Melissococcus plutonius*, the etiological agents of the American foulbrood (AFB) and European foulbrood (EFB) respectively, are bacteria that proliferate within the gut of honeybee larvae [53,54] and their EPSPS belong to the glyphosate-sensitive Class I and to the glyphosate-resistant Class II respectively (S5 Fig). Such differential sensitivity could lead to the odd hypothesis that *M*. *plutonius* could benefit from an imbalanced gut flora, favoring EFB, and in contrast, that glyphosate could reduce the development of the AFB. Future *in vivo* experiments could tell if glyphosate alters, in one way or another, the virulence of those gut pathogens.

### AMPA appeared to be no risk for the honeybee

The main metabolite of glyphosate, AMPA, that is more persistent and often more abundant than glyphosate in the environment, did not induce any significant change in the honeybee microbiota (Fig 1), although it can affect the growth of *G*. *apicola in vitro* (Fig 2). If *G*. *apicola* would include taxa with different sensitivities to glyphosate, it could be similar with AMPA. It is possible that the sensitive strain isolated in this work is scarce in the honeybee gut.

The mode of action of AMPA remains unclear. It has been suggested that, as a glycine analog, AMPA could inhibit the mammal serine hydroxymethyltransferase, or SHMT [57]. SHMTs are involved in the folate-mediated one-carbon metabolism, and thus in purine biosynthesis and in the reversible glycine to serine conversion. It has also been proposed that AMPA could act as an alanine analog that would inhibit enzymes involved in peptidoglycan biosynthesis, explaining its antimicrobial properties [58]. Genes encoding all these enzymes are present in the genomes of the species discussed in this work, including *G*. *apicola*, *S*. *alvi* and *L*. *kunkeei*. The comparison of protein sequences with those available in the UniProt database did not allow formulating hypotheses about variation in their sensitivity and the higher sensitivity of *G*. *apicola* to AMPA may as well be linked to other mechanisms.

The absence of bacterial response to AMPA *in vivo* demonstrated that glyphosate itself is the active component affecting the microbiota. Its degradation to AMPA may contrariwise reduce its effects, although the toxicity of AMPA on the honeybee physiology is unknown. In the environment, glyphosate is mainly degraded into AMPA by soil and saprophyte bacterial and fungal communities [3–5]. Such transformation would be beneficial for the honeybee by reducing the concentration of glyphosate.

## Conclusion

In the context of honeybee population decline, where combinations of environmental contaminants are suspected, the weakening of honeybee health through the exposure to xenobiotics of low toxicity may be of importance. Glyphosate has sublethal effects on the honeybee gut microbiota, changing the abundance of major bacterial taxa, especially by affecting the growth of *S*. *alvi*. The consequences of such microbial disturbance are unclear: is the honeybee able to cope with glyphosate and preserve the gut homeostasis or will future data demonstrate that this changes are linked with a functional dysbiosis?

An increasing amount of data proves that the honeybee microbiota takes its part in the response to stressors, showing that the whole holobiont, *i*.*e*. the honeybee and its microbial communities, should be considered when challenging environmental constraints.

## Supporting information

S1 Fig Cumulative proportion of surviving honeybees chronically exposed to glyphosate (A), AMPA (B), stoichiometric mixes of glyphosate and AMPA (C) in summer, or exposed to glyphosate and/or *N*. *ceranae* in winter (D). Glyphosate (GLY) and aminomethylphosphonic acid (AMPA) were added in the feeding sugar syrup, and spores of *N*. *ceranae* were orally administrated the day preceding the experiment. Control bees were not exposed to any stressor. Survival proportion was estimated using the Kaplan-Meier method. Thick curves represent the mean values from five colony replicates (n = 5) and the thin curves represent the amplitude of the standard error. Data were considered significant when reproduced in all colony replicates. Only the decrease of survival in infected bees (D) was significant in all colony replicates (Log rank χ^2^>6.7 and *p*<0.01).
(PDF)

S2 Fig Daily mean sucrose (A) and herbicide (B) consumption of honeybees chronically exposed to glyphosate and/or AMPA in summer or to glyphosate and/or *N*. *ceranae* in winter. Glyphosate (GLY) and aminomethylphosphonic acid (AMPA) were added in the feeding sugar syrup, and spores of *N*. *ceranae* were orally administrated the day preceding the experiment. Control bees were not exposed to any stressor. (A) The sugar consumption was measured daily and reported to the number of surviving bees. No significant difference was observed. (B) The mean daily glyphosate (white) and AMPA (grey) uptake was expressed in nmol/bee/d (upper scale) and in μg/bee/d of molecules (lower scales). Bars represent the 95% confidence intervals.
(PDF)

S3 Fig Principal component analyses of QPCR data (A) for control and 1.5 mM glyphosate-treated samples in summer and winter, and (B) for glyphosate- and AMPA-treated samples in summer. Analyses were performed using the normalized CQ (relative to total bacterial content). In order to avoid redundant data for the same taxa, values obtained using *Lactobacillus* Firm-5 primers as well as Neiss-F/Neiss-R and Gill-F/Gill-R were omitted. Very similar results were obtained exchanging primer pairs data for a same bacterial taxon. Using glyphosate data only on both season (A), PCA analysis showed that 91.6% of the variance were explained by two principal components that seemed mainly linked to the season and to the glyphosate treatment. However, two-ways ANOVA (not shown) did not detect significant interaction between herbicides and season. Using the data in summer (B), PCA revealed that 70.9% of the variance was explained to one single component mostly related to glyphosate treatment, *S*. *alvi* being the main variable correlated with the component.
(PDF)

S4 Fig Growth of *S*. *alvi* strains IHP.IC1 (filled triangles) and IHP.IO2 (open circles) isolated from honeybee guts in the presence of glyphosate. Strains were inoculated in HIB medium in the presence of increasing concentrations of glyphosate. After 48h of growth under anaerobic conditions, the optical density (OD) of the cultures was measured at 600 nm. Data were gathered from six independent replicates. The log of the final OD seemed correlated to the glyphosate concentration (r: correlation coefficient, r^2^: determination coefficient), suggesting a dose-dependent reduction of the *S*. *alvi* produced biomass by the herbicide.
(PDF)

S5 Fig Phylogenetic analysis (A) and conserved amino acids (B) of 5-enolpyruvylshikimate-3-phosphate synthases (EPSPS). (A) amino acid sequences were taken from a previously reported phylogenetic tree [1], and sequences of species phylogenetically related to the isolated strains were selected in GenBank. *E*. *coli* MurA sequence was used as an outgroup belonging to the EPSPS family. Sequences were aligned using Muscle and trimmed to 726 sites (amino acids 4 to 419 of *V*. *cholerae* reference sequence). Le and Gascuel model with discrete Gamma distribution and allowance for invariant sites (LG+I+G) was selected as best-fit model of protein evolution using ProtTest [2]. The Bayesian phylogenetic tree was inferred using MrBayes V3.2.6 software [3], with branch probabilities evaluated from 865 000 simulations and 15% burn-in. The consensus phylogenetic tree was built by majority greedy clustering with ≥ 0.5 support probability. The glyphosate-sensitive Class Iα and Class Iβ and the glyphosate-resistant Class II EPSPS are indicated in blue, green and red respectively. (B) Presence of the conserved amino acid residues characteristic of Classes Iα (blue), Iβ (green) and II (red) according to Light *et al*. [1] in the representative species mentioned in A. Positions are given for reference sequences from *V*. *cholerae* (Class I) and *C*. *burnetii* (Class II). Stars (*) indicate non-conserved amino acids.
(PDF)

S6 Fig Evolutionary relationships within *Snodgrassella alvi* (A) and *Gilliamella apicola* (B) species. 16S nucleotide sequences were taken from GenBank and from Motta *et al*. [1], the latter being indicated with genome accession numbers. *K*. *negevensis* (A) and *O*. *hercynius* (B) were chosen as outgroups. Sequences were aligned using ClustalW and trimmed to 957 sites (nucleotides 378 to 1334 of MH782110) for *S*. *alvi* and to 993 sites (nucleotides 382 to 1366 of MH782109) for *G*. *apicola*. When there were redundant aligned sequences, only one was kept. General Time Reversible model with discrete Gamma distribution and allowance for invariant sites (GTR+I+G) was selected as best-fit model of nucleotide substitution using jModelTest2 [2]. The phylogenetic trees were inferred using Bayesian analyses implemented in MrBayes V3.2.6 [3], with branch probabilities evaluated from 1 100 000 simulations, with 10% burn-in. Consensus phylogenetic trees were built by majority greedy clustering with ≥ 0.5 support probability. The names of species are not indicated except when other attribution has been made. The strains isolated in this work are indicated by arrows. Stars indicate sequences present in the *A*. *mellifera* gut microbiota (others are found in other *Apis* and *Bombus* species). *S*. *alvi* sequences comprising the primer sequences of Neiss-F [F1] and Neiss-R [R1] or the sequences of Beta-1009-qtF [F2] and Beta-1115-qtR [R2] are indicated in blue and red respectively. Taxa recognized by G1-459-qtF [F1] and G1-648-qtR [R1] or by Gill-F [F2] and Gill-R [R2] appear in green and yellow respectively. Primers are referenced in S1 Table.
(PDF)

S1 Table Primers used in the study. (PDF)

S2 Table Strains isolated from honeybee guts. (PDF)
