# Supplementary material for: Glyphosate, but not its metabolite AMPA, alters the honeybee gut microbiota
Source: PLoS One. 2019 Apr 16;14(4):e0215466. doi: 10.1371/journal.pone.0215466 (PMC6467416; doi:10.1371/journal.pone.0215466)
Supplement: S1 Fig — Glyphosate (GLY) and aminomethylphosphonic acid (AMPA) were added in the feeding sugar syrup, and spores of N. ceranae were orally administrated the day preceding the experiment. Control bees were not exposed to any stressor. Survival proportion was estimated using the Kaplan-Meier method. Thick curves represent the mean values from five colony replicates (n = 5) and the thin curves represent the amplitude of the standard error. Data were considered significant when reproduced in all colony replicates. Only the decrease of survival in infected bees (D) was significant in all colony replicates (Log rank χ2>6.7 and p<0.01). (PDF) [file pone.0215466.s001.pdf]

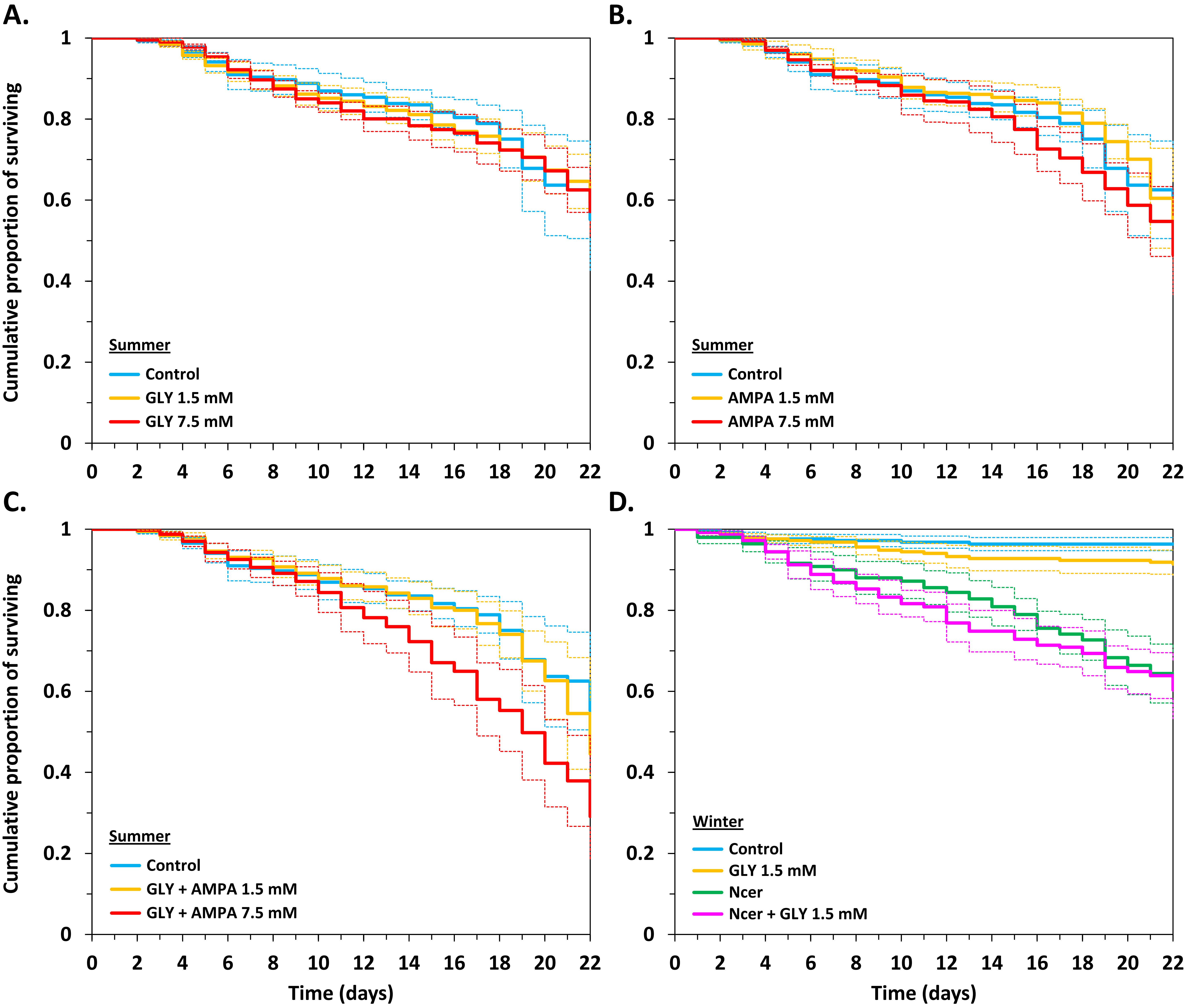

S1 Fig. Cumulative proportion of surviving honeybees chronically exposed to glyphosate (A), AMPA (B), stoichiometric mixes of glyphosate and AMPA (C) in summer, or exposed to glyphosate and/or *N. ceranae* in winter (D). Glyphosate (GLY) and aminomethylphosphonic acid (AMPA) were added in the feeding sugar syrup, and spores of *N. ceranae* were orally administrated the day preceding the experiment. Control bees were not exposed to any stressor. Survival proportion was estimated using the Kaplan-Meier method. Thick curves represent the mean values from five colony replicates (n = 5) and the thin curves represent the amplitude of the standard error. Data were considered significant when reproduced in all colony replicates. Only the decrease of survival in infected bees (D) was significant in all colony replicates (Log rank $\chi^2>6.7$ and $p<0.01$).
