# Supplementary material for: Glyphosate, but not its metabolite AMPA, alters the honeybee gut microbiota
Source: PLoS One. 2019 Apr 16;14(4):e0215466. doi: 10.1371/journal.pone.0215466 (PMC6467416; doi:10.1371/journal.pone.0215466)
Supplement: S2 Fig — Glyphosate (GLY) and aminomethylphosphonic acid (AMPA) were added in the feeding sugar syrup, and spores of N. ceranae were orally administrated the day preceding the experiment. Control bees were not exposed to any stressor. (A) The sugar consumption was measured daily and reported to the number of surviving bees. No significant difference was observed. (B) The mean daily glyphosate (white) and AMPA (grey) uptake was expressed in nmol/bee/d (upper scale) and in μg/bee/d of molecules (lower scales). Bars represent the 95% confidence intervals. (PDF) [file pone.0215466.s002.pdf]

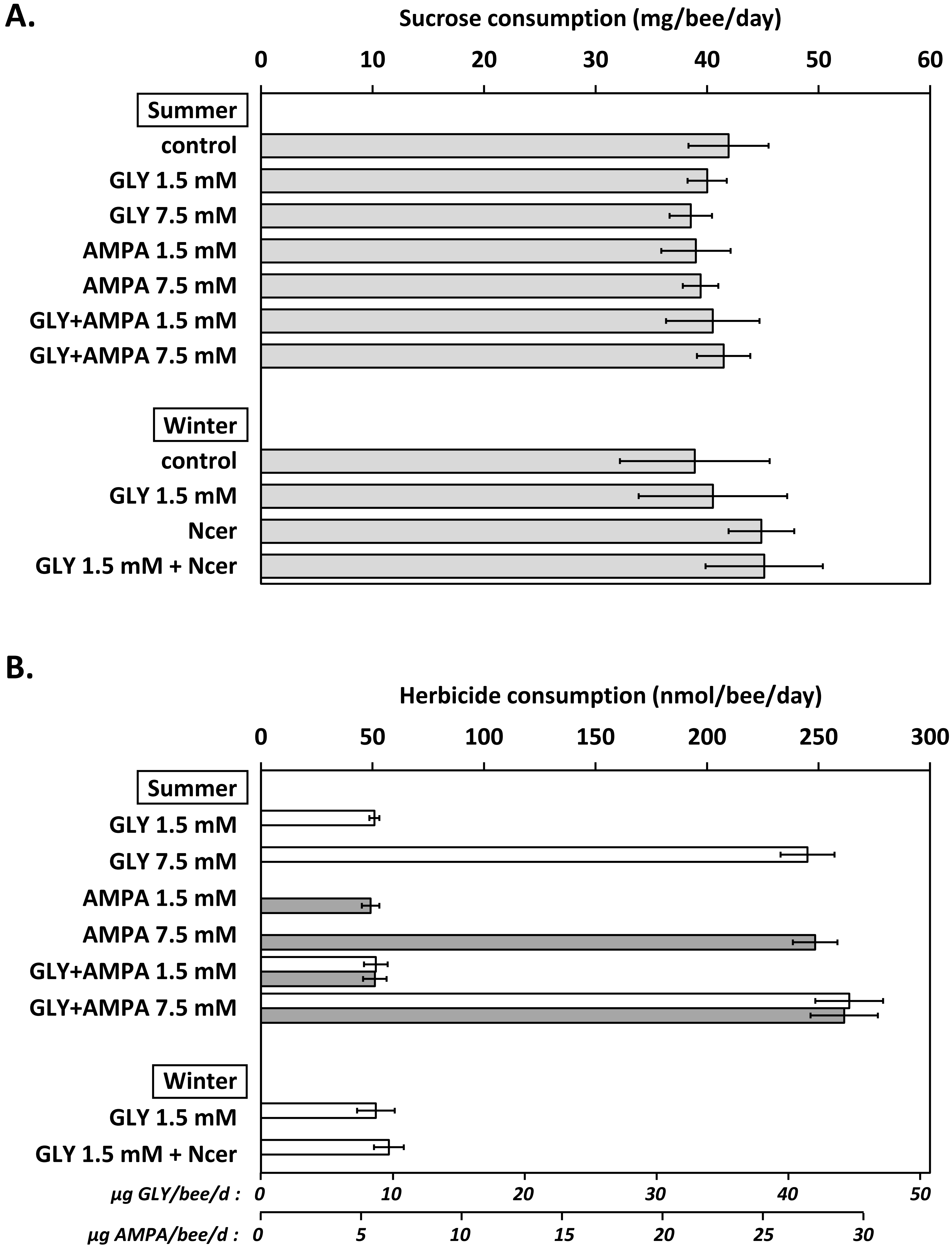

S2 Fig. Daily mean sucrose (A) and herbicide (B) consumption of honeybees chronically exposed to glyphosate and/or AMPA in summer or to glyphosate and/or *N. ceranae* in winter. Glyphosate (GLY) and aminomethylphosphonic acid (AMPA) were added in the feeding sugar syrup, and spores of *N. ceranae* were orally administrated the day preceding the experiment. Control bees were not exposed to any stressor. (A) The sugar consumption was measured daily and reported to the number of surviving bees. No significant difference was observed. (B) The mean daily glyphosate (white) and AMPA (grey) uptake was expressed in nmol/bee/d (upper scale) and in µg/bee/d of molecules (lower scales). Bars represent the 95% confidence intervals.
