# Supplementary material for: Glyphosate, but not its metabolite AMPA, alters the honeybee gut microbiota
Source: PLoS One. 2019 Apr 16;14(4):e0215466. doi: 10.1371/journal.pone.0215466 (PMC6467416; doi:10.1371/journal.pone.0215466)
Supplement: S3 Fig — Analyses were performed using the normalized CQ (relative to total bacterial content). In order to avoid redundant data for the same taxa, values obtained using Lactobacillus Firm-5 primers as well as Neiss-F/Neiss-R and Gill-F/Gill-R were omitted. Very similar results were obtained exchanging primer pairs data for a same bacterial taxon. Using glyphosate data only on both season (A), PCA analysis showed that 91.6% of the variance were explained by two principal components that seemed mainly linked to the season and to the glyphosate treatment. However, two-ways ANOVA (not shown) did not detect significant interaction between herbicides and season. Using the data in summer (B), PCA revealed that 70.9% of the variance was explained to one single component mostly related to glyphosate treatment, S. alvi being the main variable correlated with the component. (PDF) [file pone.0215466.s003.pdf]

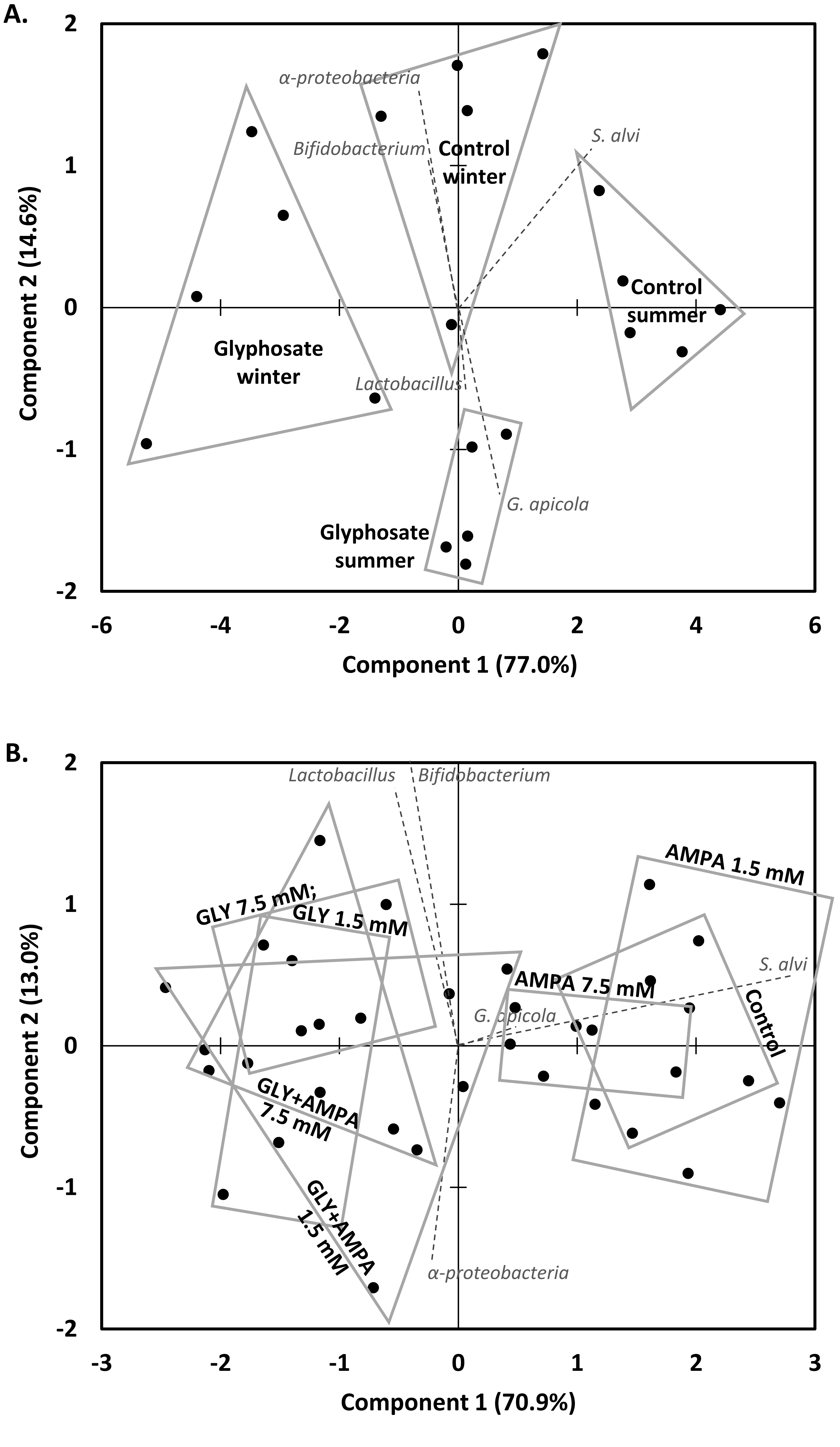

S3 Fig. Principal component analyses of QPCR data (A) for control and 1.5 mM glyphosate-treated samples in summer and winter, and (B) for glyphosate- and AMPA-treated samples in summer. Analyses were performed using the normalized CQ (relative to total bacterial content). In order to avoid redundant data for the same taxa, values obtained using *Lactobacillus* Firm-5 primers as well as Neiss-F/Neiss-R and Gill-F/Gill-R were omitted. Very similar results were obtained exchanging primer pairs data for a same bacterial taxon. Using glyphosate data only on both season (A), PCA analysis showed that 91.6% of the variance were explained by two principal components that seemed mainly linked to the season and to the glyphosate treatment. However, two-ways ANOVA (not shown) did not detect significant interaction between herbicides and season. Using the data in summer (B), PCA revealed that 70.9% of the variance was explained to one single component mostly related to glyphosate treatment, *S. alvi* being the main variable correlated with the component.
