# Supplementary material for: Glyphosate, but not its metabolite AMPA, alters the honeybee gut microbiota
Source: PLoS One. 2019 Apr 16;14(4):e0215466. doi: 10.1371/journal.pone.0215466 (PMC6467416; doi:10.1371/journal.pone.0215466)
Supplement: S4 Fig — Strains were inoculated in HIB medium in the presence of increasing concentrations of glyphosate. After 48h of growth under anaerobic conditions, the optical density (OD) of the cultures was measured at 600 nm. Data were gathered from six independent replicates. The log of the final OD seemed correlated to the glyphosate concentration (r: correlation coefficient, r2: determination coefficient), suggesting a dose-dependent reduction of the S. alvi produced biomass by the herbicide. (PDF) [file pone.0215466.s004.pdf]

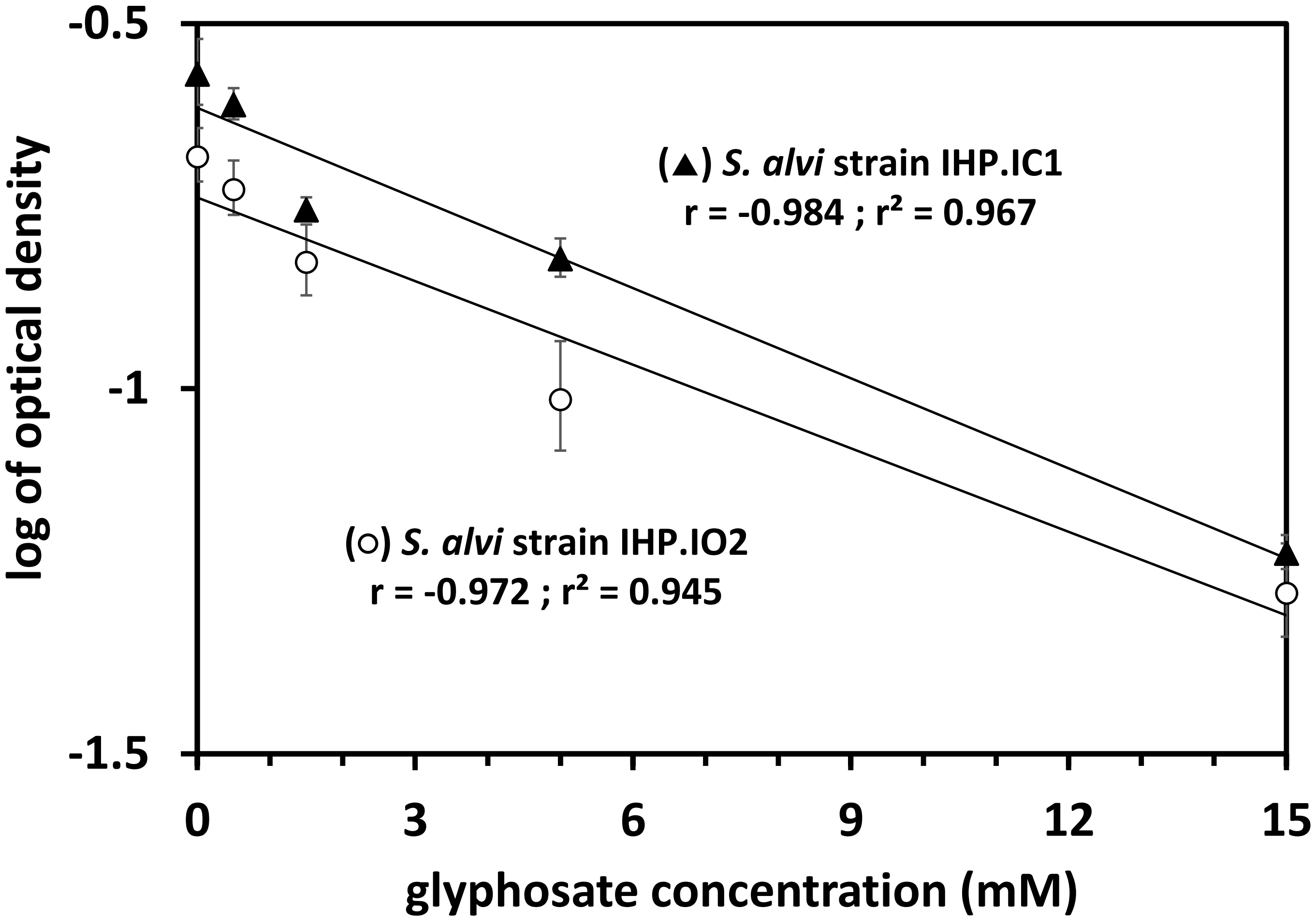

S4 Fig. Growth of *S. alvi* strains IHP.IC1 (filled triangles) and IHP.IO2 (open circles) isolated from honeybee guts in the presence of glyphosate. Strains were inoculated in HIB medium in the presence of increasing concentrations of glyphosate. After 48h of growth under anaerobic conditions, the optical density (OD) of the cultures was measured at 600 nm. Data were gathered from six independent replicates. The log of the final OD seemed correlated to the glyphosate concentration (r: correlation coefficient, r^2: determination coefficient), suggesting a dose-dependent reduction of the *S. alvi* produced biomass by the herbicide.
