# Supplementary material for: Glyphosate, but not its metabolite AMPA, alters the honeybee gut microbiota
Source: PLoS One. 2019 Apr 16;14(4):e0215466. doi: 10.1371/journal.pone.0215466 (PMC6467416; doi:10.1371/journal.pone.0215466)
Supplement: S5 Fig — (A) amino acid sequences were taken from a previously reported phylogenetic tree [1], and sequences of species phylogenetically related to the isolated strains were selected in GenBank. E. coli MurA sequence was used as an outgroup belonging to the EPSPS family. Sequences were aligned using Muscle and trimmed to 726 sites (amino acids 4 to 419 of V. cholerae reference sequence). Le and Gascuel model with discrete Gamma distribution and allowance for invariant sites (LG+I+G) was selected as best-fit model of protein evolution using ProtTest [2]. The Bayesian phylogenetic tree was inferred using MrBayes V3.2.6 software [3], with branch probabilities evaluated from 865 000 simulations and 15% burn-in. The consensus phylogenetic tree was built by majority greedy clustering with ≥ 0.5 support probability. The glyphosate-sensitive Class Iα and Class Iβ and the glyphosate-resistant Class II EPSPS are indicated in blue, green and red respectively. (B) Presence of the conserved amino acid residues characteristic of Classes Iα (blue), Iβ (green) and II (red) according to Light et al. [1] in the representative species mentioned in A. Positions are given for reference sequences from V. cholerae (Class I) and C. burnetii (Class II). Stars (*) indicate non-conserved amino acids. (PDF) [file pone.0215466.s005.pdf]

A.

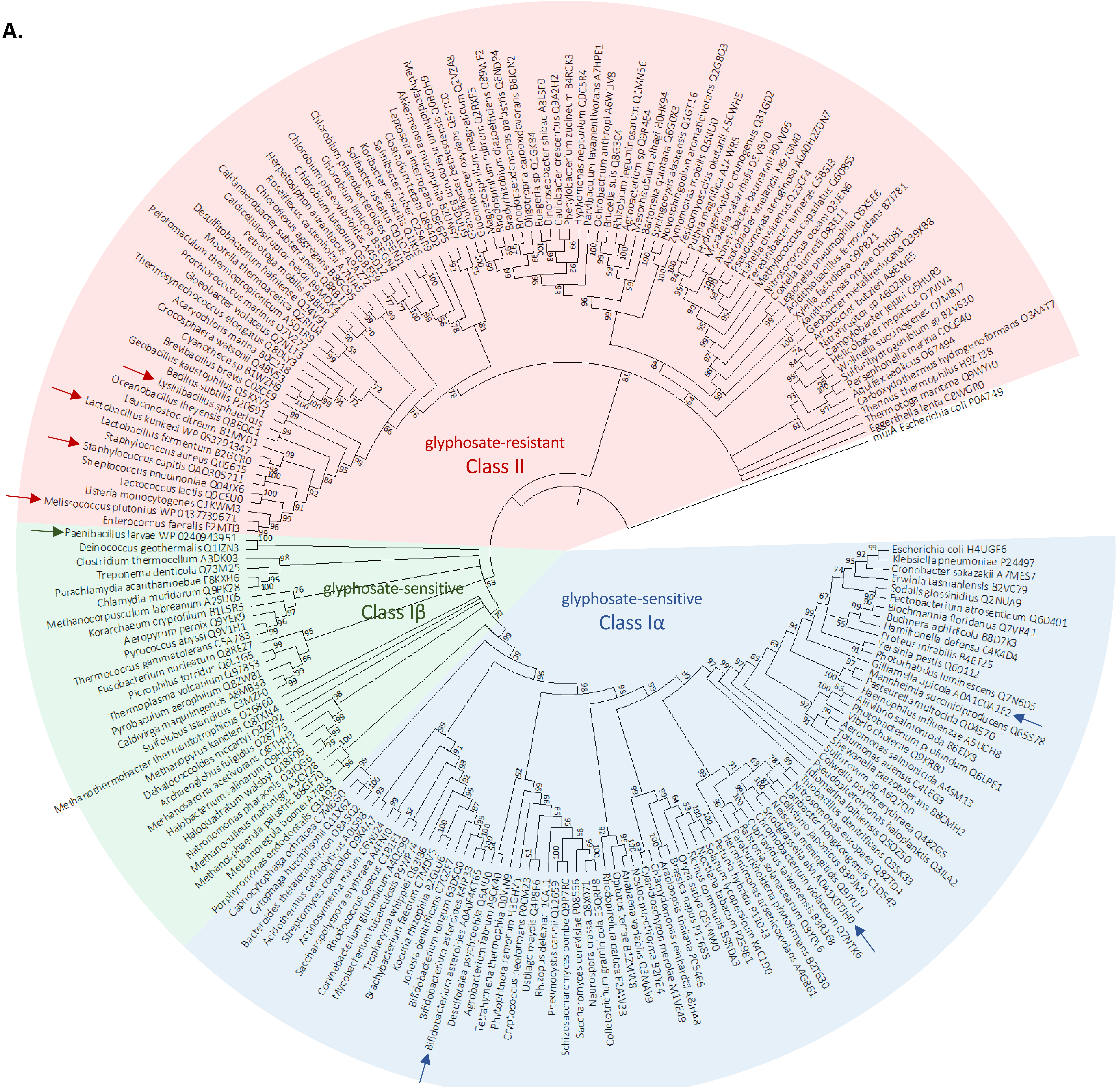

B.

Position																																												
Class I (*Vibtrio cholerae*)	21	22	23	26	27	49	94	96	97	100	104	117	118	124	125	128	132	133	150	170	171	172	174	199	203	205	241	243	245	269	272	314	315	337	341	342	345	357	385	386	387	412	413	415
Class II (*Coxiella burnetti*)	20	21	22	25	26	48	93	95	96	99	103	116	117	123	124	127	131	132	147	167	168	169	171	194	198	200	231	233	235	260	263	315	316	338	342	343	346	358	385	386	387	413	414	416
**Residue Class Iα**	S	K	S	*	R	D	*	G	T	R	*	*	*	R	P	*	*	*	P	S	S	Q	*	*	*	T	E	D	*	*	Q	*	*	*	*	E	R	G	D	H	R	K	*	P
Snodgrassella alvi	S	K	S	N	R	D	N	G	T	R	A	G	V	R	P	D	A	L	P	S	S	Q	L	K	A	T	E	D	S	Q	Q	D	A	S	K	E	R	G	D	H	R	K	T	P
Gilliamella apicola	S	K	S	N	R	D	N	G	T	R	A	G	E	R	P	H	A	L	P	S	S	Q	L	K	D	T	E	D	S	K	Q	D	A	N	K	E	R	G	D	H	R	K	T	P
Bifidobacterium asteroides	S	K	S	N	R	D	L	G	T	R	G	G	D	R	P	P	G	L	P	S	S	Q	L	L	T	T	E	D	S	D	Q	E	A	H	H	E	R	G	D	H	R	K	T	P
**Residue Class Iβ**	S	K	*	*	R	*	*	*	*	*	*	*	*	R	P	*	*	*	*	S	S	Q	*	*	*	T	*	D	*	*	*	D	*	*	K	E	R	G	D	H	R	*	*	*
Paenibacillus larvae	S	K	N	T	R	D	N	G	A	R	S	Y	P	R	P	D	A	L	P	S	S	Q	L	K	G	T	Q	D	P	N	Q	D	A	N	K	E	R	G	D	H	R	K	S	P
**Residue Class II**	D	K	S	H	R	D	N	*	*	R	G	G	D	R	P	R	P	L	*	S	A	Q	K	R	E	*	P	D	S	N	R	D	E	E	K	E	R	*	D	H	R	*	S	P
Lactobacillus kunkeei	D	K	S	H	R	D	N	G	T	R	G	G	D	R	P	R	P	L	P	S	A	Q	K	R	E	M	P	D	S	N	R	D	E	E	K	E	R	G	D	H	R	I	S	P
Lysinibacillus sphaericus	D	K	S	H	R	D	N	G	T	R	G	G	D	R	P	R	P	L	P	S	A	Q	K	R	E	M	P	D	S	N	R	D	E	E	K	E	R	G	D	H	R	V	S	P
Melissococcus plutonius	D	K	S	H	R	D	N	G	T	R	G	G	D	R	P	R	P	L	P	S	A	Q	K	R	E	M	P	D	S	N	R	D	E	E	K	E	R	G	D	H	R	V	S	P
Staphylococcus capitis	D	K	S	H	R	D	N	G	T	R	G	G	D	R	P	R	P	L	P	S	A	Q	K	R	E	M	P	D	S	N	R	D	E	E	K	E	R	G	D	H	R	V	S	P

S5 Fig. Phylogenetic analysis (A) and conserved amino acids (B) of 5-enolpyruvylshikimate-3-phosphate synthases (EPSPS). (A) amino acid sequences were taken from a previously reported phylogenetic tree [1], and sequences of species phylogenetically related to the isolated strains were selected in GenBank. *E. coli* MurA sequence was used as an outgroup belonging to the EPSPS family. Sequences were aligned using Muscle and trimmed to 726 sites (amino acids 4 to 419 of *V. cholerae* reference sequence). Le and Gascuel model with discrete Gamma distribution and allowance for invariant sites (LG+I+G) was selected as best-fit model of protein evolution using ProtTest [2]. The Bayesian phylogenetic tree was inferred using MrBayes V3.2.6 software [3], with branch probabilities evaluated from 865 000 simulations and 15% burn-in. The consensus phylogenetic tree was built by majority greedy clustering with ≥ 0.5 support probability. The glyphosate-sensitive Class Iα and Class Iβ and the glyphosate-resistant Class II EPSPS are indicated in blue, green and red respectively. (B) Presence of the conserved amino acid residues characteristic of Classes Iα (blue), Iβ (green) and II (red) according to Light *et al.* [1] in the representative species mentioned in A. Positions are given for reference sequences from *V. cholerae* (Class I) and *C. burnetii* (Class II). Stars (*) indicate non-conserved amino acids.

References :

1. Light SH, Krishna SN, Minasov G, Anderson WF. An unusual cation-binding site and distinct domain-domain interactions distinguish Class II enolpyruvylshikimate-3-phosphate synthases. Biochemistry. 2016;55: 1239-1245.
2. Darriba D, Taboada GL, Doallo R, Posada D. ProtTest 3: fast selection of best-fit models of protein evolution. Bioinformatics. 2011;27: 1164-1165.
3. Ronquist F, Teslenko M, van der Mark P, Ayres DL, Darling A, Höhna S, Larget B, Liu L, Suchard MA, Huelsenbeck JP. Efficient Bayesian phylogenetic inference and model choice across a large model space. Systematic Biology. 2012;61: 539-542.
