# Supplementary material for: Glyphosate, but not its metabolite AMPA, alters the honeybee gut microbiota
Source: PLoS One. 2019 Apr 16;14(4):e0215466. doi: 10.1371/journal.pone.0215466 (PMC6467416; doi:10.1371/journal.pone.0215466)
Supplement: S6 Fig — 16S nucleotide sequences were taken from GenBank and from Motta et al. [1], the latter being indicated with genome accession numbers. K. negevensis (A) and O. hercynius (B) were chosen as outgroups. Sequences were aligned using ClustalW and trimmed to 957 sites (nucleotides 378 to 1334 of MH782110) for S. alvi and to 993 sites (nucleotides 382 to 1366 of MH782109) for G. apicola. When there were redundant aligned sequences, only one was kept. General Time Reversible model with discrete Gamma distribution and allowance for invariant sites (GTR+I+G) was selected as best-fit model of nucleotide substitution using jModelTest2 [2]. The phylogenetic trees were inferred using Bayesian analyses implemented in MrBayes V3.2.6 [3], with branch probabilities evaluated from 1 100 000 simulations, with 10% burn-in. Consensus phylogenetic trees were built by majority greedy clustering with ≥ 0.5 support probability. The names of species are not indicated except when other attribution has been made. The strains isolated in this work are indicated by arrows. Stars indicate sequences present in the A. mellifera gut microbiota (others are found in other Apis and Bombus species). S. alvi sequences comprising the primer sequences of Neiss-F [F1] and Neiss-R [R1] or the sequences of Beta-1009-qtF [F2] and Beta-1115-qtR [R2] are indicated in blue and red respectively. Taxa recognized by G1-459-qtF [F1] and G1-648-qtR [R1] or by Gill-F [F2] and Gill-R [R2] appear in green and yellow respectively. Primers are referenced in S1 Table. (PDF) [file pone.0215466.s006.pdf]

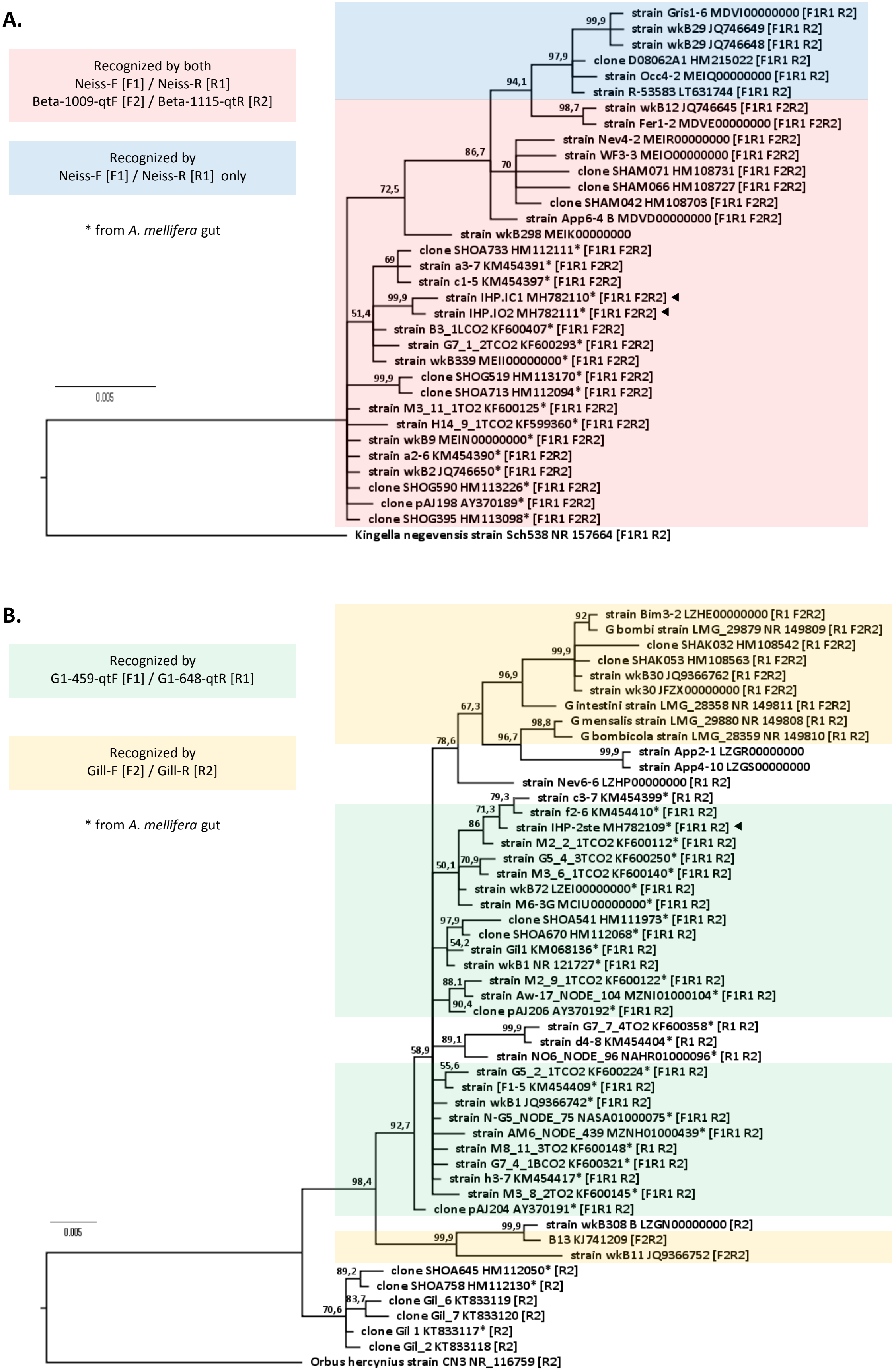


**S6 Fig. Evolutionary relationships within *Snodgrassella alvi* (A) and *Gilliamella apicola* (B) species.** 16S nucleotide sequences were taken from GenBank and from Motta *et al*. [1], the latter being indicated with genome accession numbers. *K. negevensis* (A) and *O. hercynius* (B) were chosen as outgroups. Sequences were aligned using ClustalW and trimmed to 957 sites (nucleotides 378 to 1334 of MH782110) for *S. alvi* and to 993 sites (nucleotides 382 to 1366 of MH782109) for *G. apicola*. When there were redundant aligned sequences, only one was kept. General Time Reversible model with discrete Gamma distribution and allowance for invariant sites (GTR+I+G) was selected as best-fit model of nucleotide substitution using jModelTest2 [2]. The phylogenetic trees were inferred using Bayesian analyses implemented in MrBayes V3.2.6 [3], with branch probabilities evaluated from 1 100 000 simulations, with 10% burn-in. Consensus phylogenetic trees were built by majority greedy clustering with ≥ 0.5 support probability. The names of species are not indicated except when other attribution has been made. The strains isolated in this work are indicated by arrows. Stars indicate sequences present in the *A. mellifera* gut microbiota (others are found in other *Apis* and *Bombus* species). *S. alvi* sequences comprising the primer sequences of Neiss-F [F1] and Neiss-R [R1] or the sequences of Beta-1009-qtF [F2] and Beta-1115-qtR [R2] are indicated in blue and red respectively. Taxa recognized by G1-459-qtF [F1] and G1-648-qtR [R1] or by Gill-F [F2] and Gill-R [R2] appear in green and yellow respectively. Primers are referenced in S1 Table.

References


1. Motta EVS, Raymann K, Moran NA. Glyphosate perturbs the gut microbiota of honey bees. *Proc Natl Acad Sci USA*. 2018;115: 10305-10310.
2. Posada D. jModelTest: phylogenetic model averaging. Mol Biol Evol. 2008;25: 1253-1256.
3. Ronquist F, Teslenko M, van der Mark P, Ayres DL, Darling A, Höhna S, Larget B, Liu L, Suchard MA, Huelsenbeck JP. Efficient Bayesian phylogenetic inference and model choice across a large model space. Systematic Biology. 2012;61: 539-542.
