# Supplementary material for: Glyphosate, but not its metabolite AMPA, alters the honeybee gut microbiota
Source: PLoS One. 2019 Apr 16;14(4):e0215466. doi: 10.1371/journal.pone.0215466 (PMC6467416; doi:10.1371/journal.pone.0215466)
Supplement: S1 Table — (PDF) [file pone.0215466.s007.pdf]

**S1 Table. Primers used in the study**

| Targeted group | Primer pairs and sequences (5'→3') | Reference |
|---|---|---|
| **Primers amplifying SSU fragments for QPCR analyses** | | |
| Bacteria | 341F CCTACGGGAGGCAGCAG<br>534R ATTACCGCGGCTGCTGGCA | Bru D, Martin-Laurent F, Philippot L. Quantification of the detrimental effect of a single primer-template mismatch by real-time PCR using the 16S rRNA gene as an example. Appl Environ Microbiol. 2008;74: 1660-1663 |
| Bacteria | BAC338F ACTCCTACGGGAGGCAG<br>BAC805R GACTACCAGGGTATCTAATCC | Yu Y, Lee C, Kim J, Hwang S. Group-specific primer and probe sets to detect methanogenic communities using quantitative real-time polymerase chain reaction. Biotechnol Bioeng. 2005;89: 670-679 |
| *Bifidobacterium* spp. | Bifi-F2 TCGCGTCYGGTGTGAAAG<br>Bifi-R2 CCACATCCAGCRTCCAC | Rinttilä T, Kassinen A, Malinen E, Krogius L, Palva A. Development of an extensive set of 16S rDNA-targeted primers for quantification of pathogenic and indigenous bacteria in faecal samples by real-time PCR. J Appl Microbiol. 2004;97: 1166-1177 |
| *Lactobacillus* Firm-5 | Lact-F TAACGCATTAAGCACTCC<br>Lact-R GCTGGCAACTAATAATAAGG | Li J, Qin H, Wu J, Sadd BM, Wang X, Evans JD, Peng W, Chen Y. The prevalence of parasites and pathogens in Asian honeybees *Apis cerana* in China. PLoS One. 2012;7: e47955 |
| *Lactobacillus* spp. | Lact-F2 AGCAGTAGGGAATCTTCCA<br>Lact-R2 CACCGCTACACATGGAG | Rinttilä T, Kassinen A, Malinen E, Krogius L, Palva A. Development of an extensive set of 16S rDNA-targeted primers for quantification of pathogenic and indigenous bacteria in faecal samples by real-time PCR. J Appl Microbiol. 2004;97: 1166-1177 |
| $\alpha$-proteobacteria | a682F CDAGTGTAGAGGTGAAATT<br>908aR CCCCGTCAATTCCTTTGAGTT | Bacchetti de Gregoris T, Aldred N, Clare AS, Burgess JG. Improvement of phylum- and class-specific primers for real-time PCR quantification of bacterial taxa. J Microbiol Methods. 2011;86: 351-356 |
| *Snodgrassella alvi* | Neiss-F AAGCGGTGGATGATGTGG<br>Neiss-R TGATGGCAACTAATGACAAGG | Li J, Qin H, Wu J, Sadd BM, Wang X, Evans JD, Peng W, Chen Y. The prevalence of parasites and pathogens in Asian honeybees *Apis cerana* in China. PLoS One. 2012;7: e47955 |
| *Snodgrassella alvi* | Beta-1009-qtF CTTAGAGATAGGAGAGTG<br>Beta-1115-qtR AATGATGGCAACTAATGACAA | Martinson VG, Moy J, Moran NA. Establishment of characteristic gut bacteria during development of the honeybee worker. Appl Environ Microbiol. 2012;78: 2830-2840 |
| *Gilliamella apicola* | G1-459-qtF GTATCTAATAGGTGCATCAATT<br>G1-648-qtR TCCTCTACAATACTCTAGTT | Martinson VG, Moy J, Moran NA. Establishment of characteristic gut bacteria during development of the honeybee worker. Appl Environ Microbiol. 2012;78: 2830-2840 |
| *Gilliamella apicola* | Gill-F CCTTTGTTGCCATCGATTAGG<br>Gill-R GACATTCTGATTCACGATTACTAGC | Xu LL, Wu J, Guo J, Li JL. Dynamic variation of symbionts in Bumblebees during hosts growth and development. *Sci. Agric. Sinica*. 2014;47: 2030–2037 |
| **Primers amplifying the SSU for sequencing** | | |
| Bacteria | 27f-YM AGAGTTTGATYMTGGCTCAG<br>27f-Bif AGGGTTCGATTCTGGCTCAG<br>1492r TACCTTGTTAYGACTT | Frank JA, Reich CI, Sharma S, Weisbaum JS, Wilson BA, Olsen GJ. Critical evaluation of two primers commonly used for amplification of bacterial 16S rRNA genes. Appl Environ Microbiol. 2008;74: 2461-2470 |
