# Supplementary material for: Glyphosate, but not its metabolite AMPA, alters the honeybee gut microbiota
Source: PLoS One. 2019 Apr 16;14(4):e0215466. doi: 10.1371/journal.pone.0215466 (PMC6467416; doi:10.1371/journal.pone.0215466)
Supplement: S2 Table — (PDF) [file pone.0215466.s008.pdf]

S2 Table. Strains isolated from honeybee guts

Organism	Isolated from	16S sequence accession number	Phylum or class, order	Media allowing growth [a]	Growth under O_2	Cell morphology
Gilliamella apicola strain IHP.2ste	bee #2	MH782109	γ-proteobacteria, Orbales	<u>HIB</u>	no	rod-shaped, Gram -
Snodgrassella alvi strain IHP.IC1	bee #5	MH782110	β-proteobacteria, Neisseriales	<u>HIB</u>,LB	no	rod-shaped, Gram -
Snodgrassella alvi strain IHP.IO2	bee #6	MH782111				
Bifidobacterium sp. strain IHP.3T2CH	bee #3	MH782112	Actinobacteria, Bifidobacteriales	<u>HIB</u>,MRS	+	rod-shaped, Gram +
Bifidobacterium sp. strain IHP.9OP	bee #9	MH782113				
Bifidobacterium sp. strain IHP.IC3	bee #5	MH782114				
Lactobacillus kunkeei strain IHP.7AA	bee #7	MH782115	Firmicutes, Lactobacillales	HIB,<u>MRS</u>	++	rod-shaped, Gram +
Lactobacillus kunkeei strain IHP.B1	bee #1	MH782116				
Lysinibacillus sp. strain IHP.3T2B	bee #3	MH782117	Firmicutes, Bacillales	<u>HIB</u>,MRS,LB	+++	rod-shaped, Gram + [b]
Lysinibacillus sp. strain IHP.4GCCB	bee #4	MH782118				
Staphylococcus sp. strain IHP.4GCCA	bee #4	MH782119	Firmicutes, Bacillales	<u>HIB</u>,MRS,LB	+	cocci, Gram +
Staphylococcus sp. strain IHP.B3	bee #1	MH782120				
Hafnia sp. strain IHP.92DB [c]	bee #9	MH782121	γ-proteobacteria, Enterobacterales	HIB,<u>MRS</u>,LB	+++	rod-shape, Gram -

[a] Medium with the best growth is underlined. HIB: Heart Infusion Broth. MRS: De Man, Rogosa and Sharpe (here supplemented with fructose and cystein). LB: Lysogeny Broth

[b] Strains forming big bacilli with central Gram positive coloration but lighter ends

[c] Colonies grown on agar media had a "flowery" smell
